# Supplementary material for: Unravelling rechargeable zinc-copper batteries by a chloride shuttle in a biphasic electrolyte
Source: Nat Commun. 2023 Apr 24;14:2349. doi: 10.1038/s41467-023-37642-2 (PMC10125991; doi:10.1038/s41467-023-37642-2)
Supplement: Supplementary file 1 — Supplementary Information [file 41467_2023_37642_MOESM1_ESM.pdf]

Supplementary Information

Unravelling rechargeable zinc-copper batteries by a chloride shuttle in a biphasic electrolyte

Chen Xu[1], Chengjun Lei[1], Jinye Li[1], Xin He[1], Pengjie Jiang[1], Huijian Wang[1], Tingting Liu[1] and Xiao Liang[1*]

1 State Key Laboratory of Chem/Biosensing and Chemometrics, College of Chemistry and Chemical Engineering, Hunan University, Changsha, 410082, P.R. China.

* Corresponding author: xliang@hnu.edu.cn

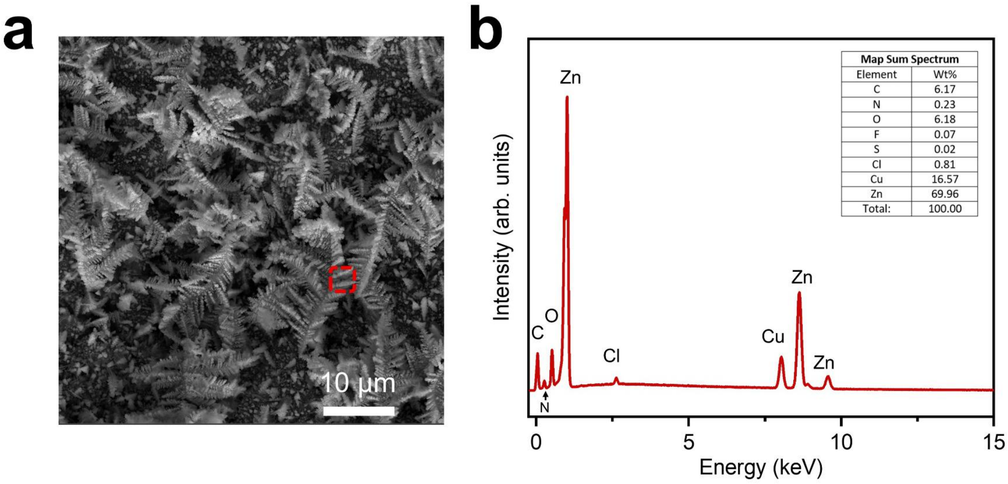

Supplementary Fig. 1 (a) The SEM images of Zn anode after 2 cycles in 0.5 m $CuCl_2$ aqueous solutions/[bmmim][Tf_2N] biphasic electrolyte, and **(b)** EDS elemental analysis for the selected area in (a).

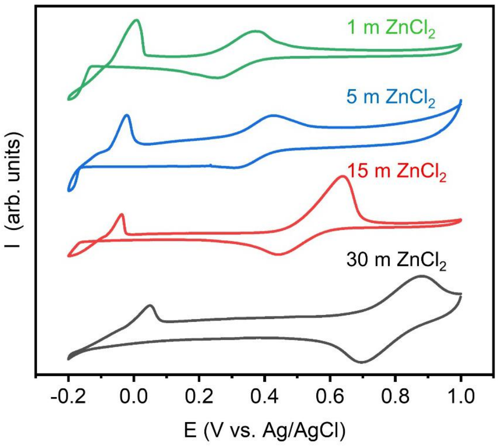

Supplementary Fig. 2 The CV curves of the $CuCl_2$ electrode in $ZnCl_2$ solutions with various concentrations.

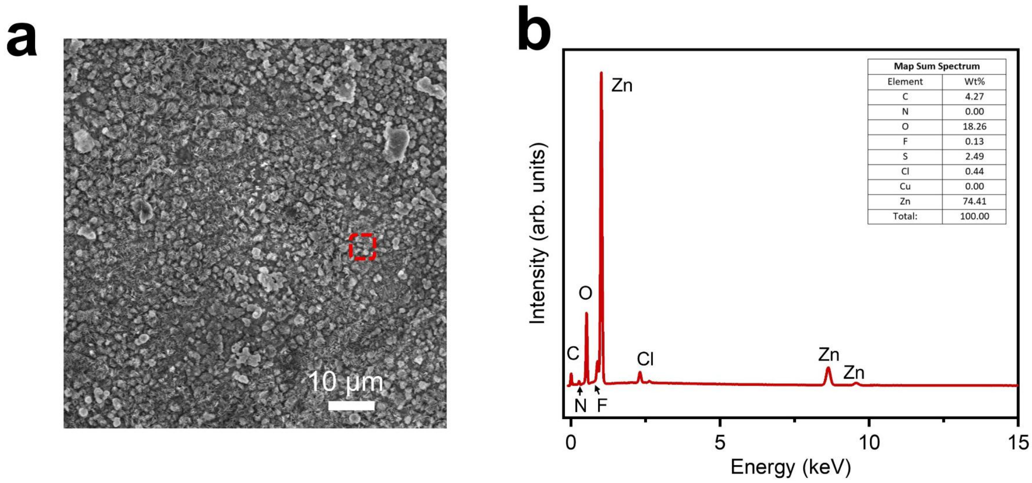

Supplementary Fig. 3 (a) The SEM images of Zn anode after 2 cycles in 15 m $ZnCl_2$ biphasic electrolyte at 200 mAh g^{-1} between 1.6 – 0.4 V, and **(b)** EDS elemental analysis for the selected area in (a).

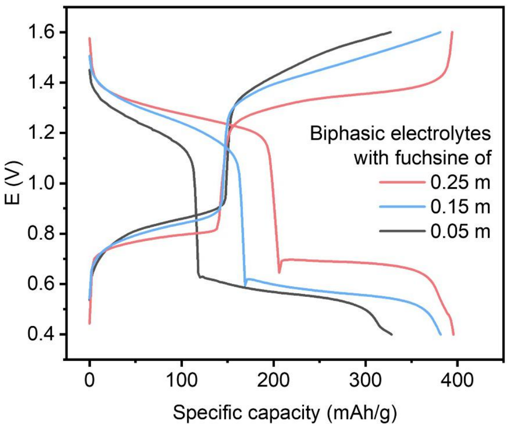

Supplementary Fig. 4 The overpotential of the Zn-Cu cells is increased along with the decrease of the fuchsine concentration. The current density was 200 mA g^{-1} (0.5C).

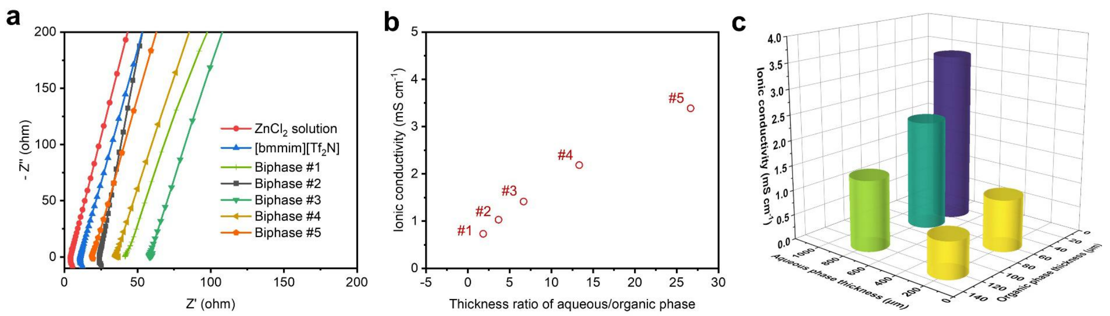

Supplementary Fig. 5 **(a)** The EIS profiles of the 15 m $ZnCl_2$ solution, [bmmim][Tf_2N], and the biphasic electrolytes. The thickness of the homogenous solutions are d_{ZnCl2}=800 μm and $d_{[bmmim][Tf2N]}$=60 μm, respectively. For the biphasic electrolyte, the $d_{biphasic\ system}$ is calculated with various combination of d_{ZnCl2} + $d_{[bmmim][Tf2N]}$, d#1= 219 + 122 μm; d#2= 220 + 60 μm; d#3= 802 + 122 μm; d#4= 800 + 62 μm; d#5= 801 + 30 μm). **(b)** The calculated ionic conductivity of the various biphasic electrolytes. (c) Three-dimensional bar graph showing ionic conductivity (vertical axis) in relation to the thickness of aqueous phase (left diagonal axis) and organic phase (right diagonal axis).

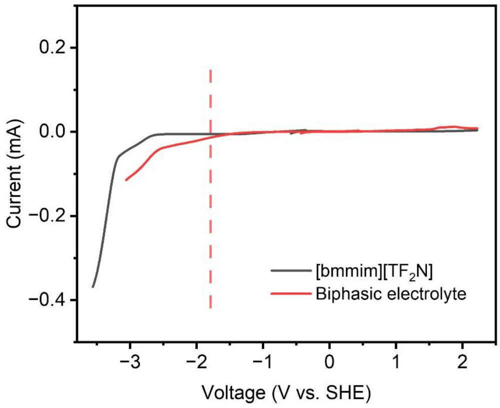

Supplementary Fig. 6 The LSV curves of the [bmmim][Tf_2N] ionic liquid and the organic phase of the biphasic electrolyte. The scan rate was set at 0.5 mV s^{-1}.

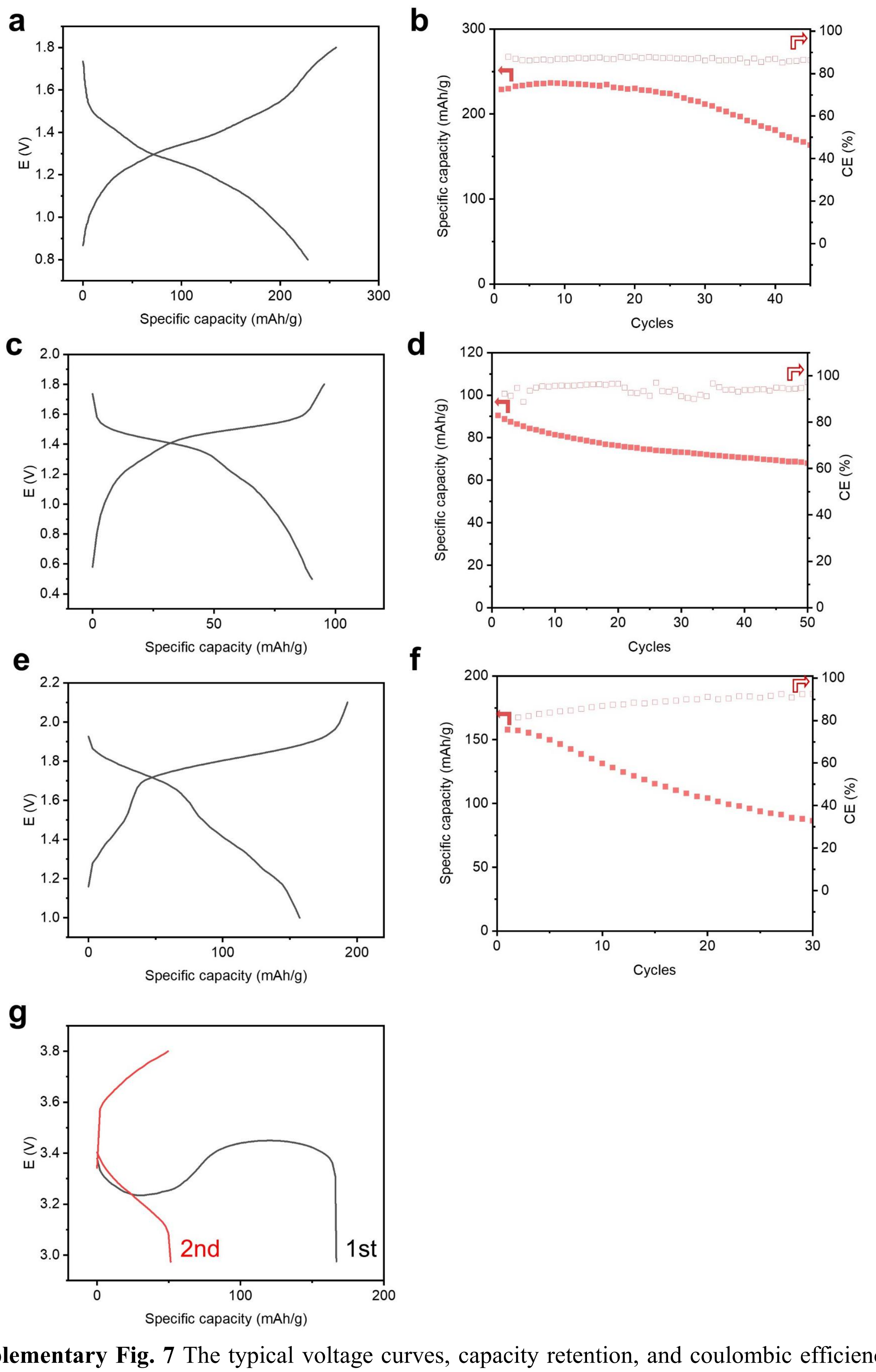

Supplementary Fig. 7 The typical voltage curves, capacity retention, and coulombic efficiencies of the batteries with $ZnCl_2$ based biphasic electrolyte at current densities of 200 mA g^{-1}. **(a, b)** $NiCl_2$ electrode in 20 m $ZnCl_2$ biphasic electrolyte, **(c, d)** $FeCl_3$ electrode in 20 m $ZnCl_2$ biphasic electrolyte, **(e, f)** V_2O_5 electrode in 10 m $ZnCl_2$ biphasic electrolyte (1 m H_2SO_4 was added in $ZnCl_2$ solution to dissolve vanadium oxide) with zinc metal anode, and **(g)** $CuCl_2$ electrode in 15 m $ZnCl_2$ based biphasic electrolyte with Li metal anode.

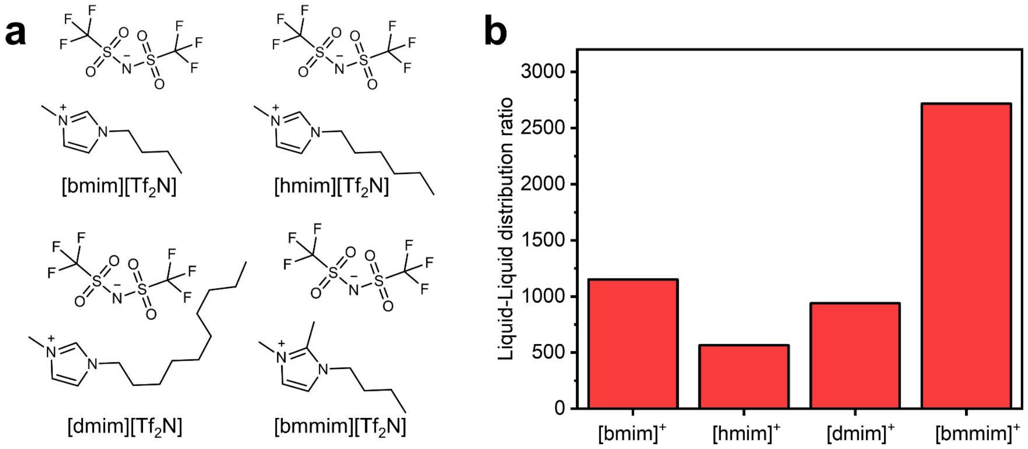

Supplementary Fig. 8 (a) The chemical structures of the Tf_2N based ILs, and **(b)** the influence of the cations to the distribution of Cu^{2+} between aqueous and organic phase.

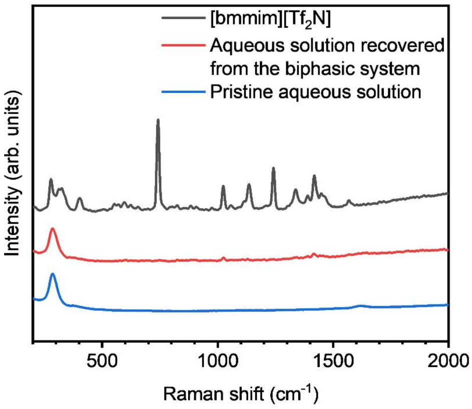

Supplementary Fig. 9 The Raman spectra of the 15 m $ZnCl_2$ aqueous solution before and after equilibrated with the [bmmim][Tf_2N] phase.

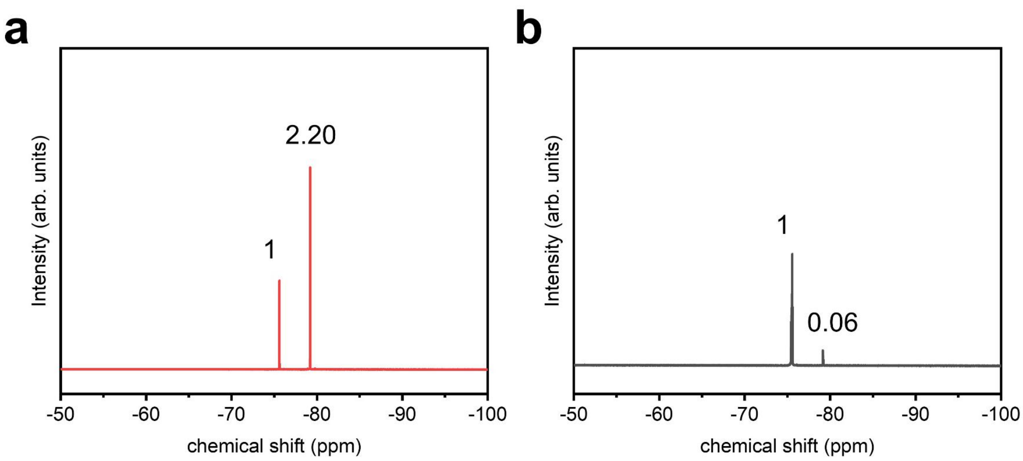

Supplementary Fig. 10 The ^{19}F NMR spectra of **(a)** pure water and **(b)** 15 m $ZnCl_2$ solution after equlibrated with the [bmmim][Tf_2N] phase. The distribution of Tf_2N^- in the aqueous phase were determined by the ^{19}F NMR with interior label of trifluoroacetic acid (-76.5 ppm). The Tf_2N^- signal is generated (-79 ppm) and its molarity is determined against the interior label. The population of Tf_2N^- in the pure water after combination with [bmmim][Tf_2N] is about 36 times higher than that in 15 m $ZnCl_2$ solution.

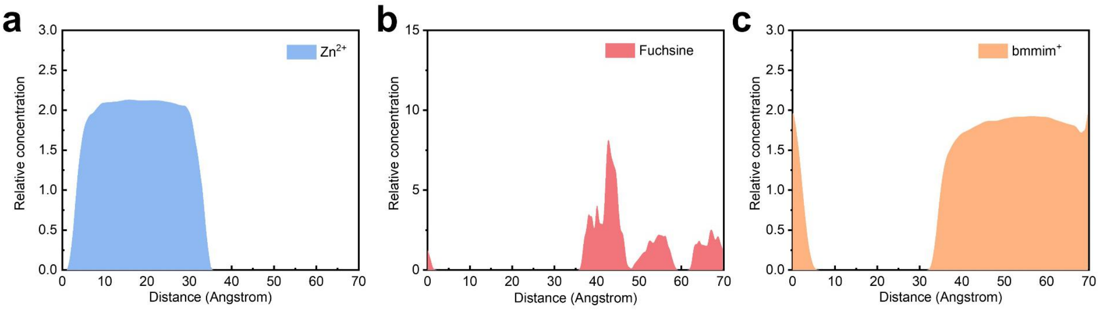

Supplementary Fig. 11 The density profiles for **(a)** Zn^{2+}, **(b)** Fuchsine, and **(c)** [bmmim]$^+$ in the interface of the biphasic electrolyte obtained by molecular dynamics simulations.

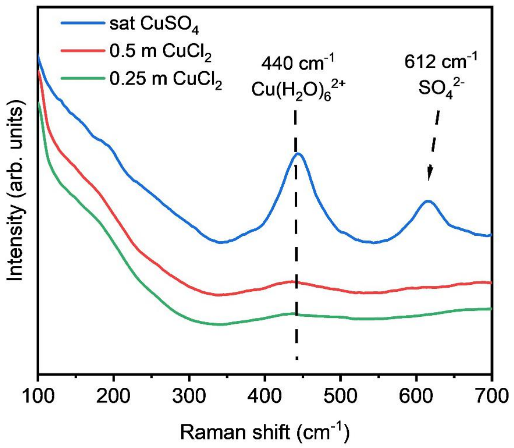

Supplementary Fig. 12 The Raman spectra of $CuCl_2$ and $CuSO_4$ aqueous solutions.

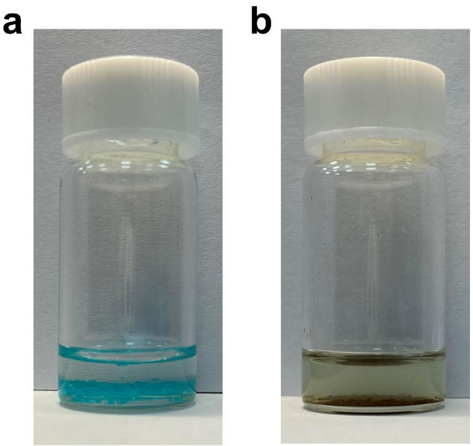

Supplementary Fig. 13 The optical photographs of 0.125 mmol **(a)** $CuCl_2 \cdot 2H_2O$ and **(b)** $CuCl_2$ solids immersed in the [bmmim][Tf_2N] phase (1 mL) to show their solubility.

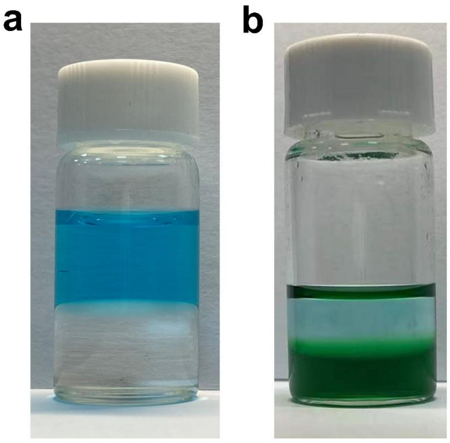

Supplementary Fig. 14 The optical photographs of the self-stratification between [bmmim][Tf_2N] and **(a)** 0.5 m $CuCl_2$ solution, **(b)** 5 m $CuCl_2$ solution.

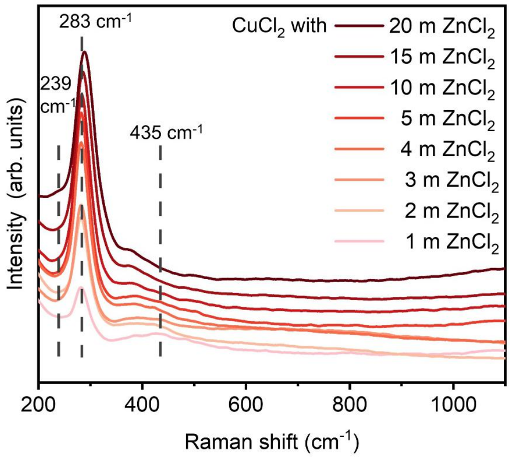

Supplementary Fig. 15 The Raman spectra of the low wavenumber regions of 1 m $CuCl_2$ in various of $ZnCl_2$ aqueous solutions.

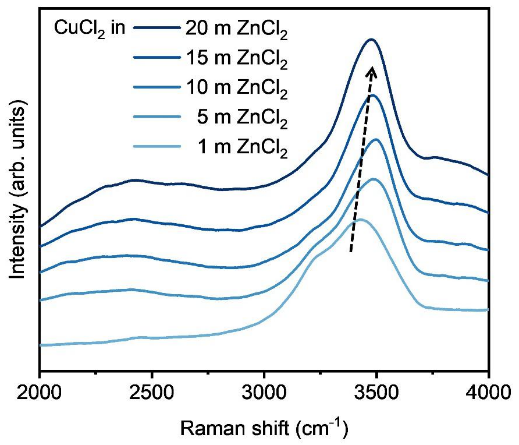

Supplementary Fig. 16 The Raman spectra of the high wavenumber region of 1 m $CuCl_2$ in various of $ZnCl_2$ aqueous solutions.

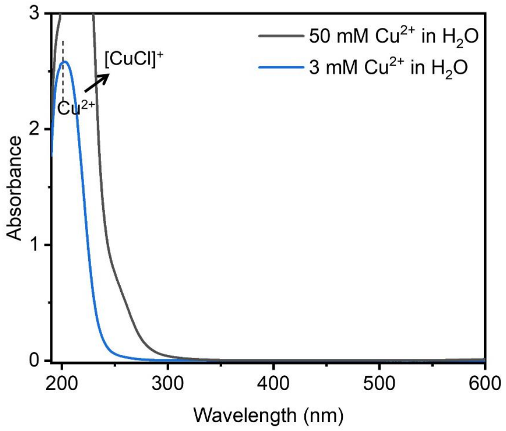

Supplementary Fig. 17 The UV-vis absorbance of cupric ion in H_2O.

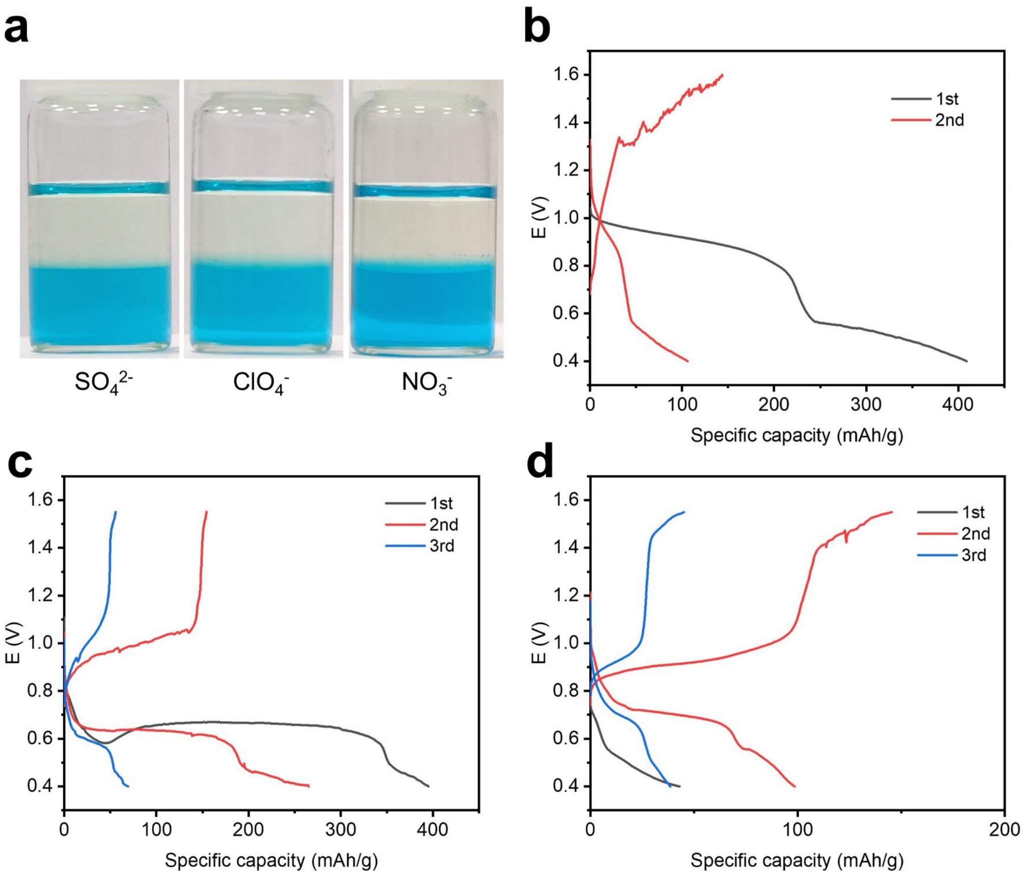

Supplementary Fig. 18 (a) Stratification between [bmmim][Tf$_2$N] ionic liquid with the aqueous phase of 4 m $ZnSO_4$ + 1 m $CuCl_2$, 5 m $Zn(ClO_4)_2$ + 1 m $CuCl_2$, and 5 m $Zn(NO_3)_2$ + 1 m $CuCl_2$ solution. Fast degradation cells with **(b)** 4 m $ZnSO_4$, **(c)** 4 m $Zn(ClO_4)_2$, and **(d)** 5 m $Zn(NO_3)_2$ as the aqueous phase of the bi-phasic electrolyte.

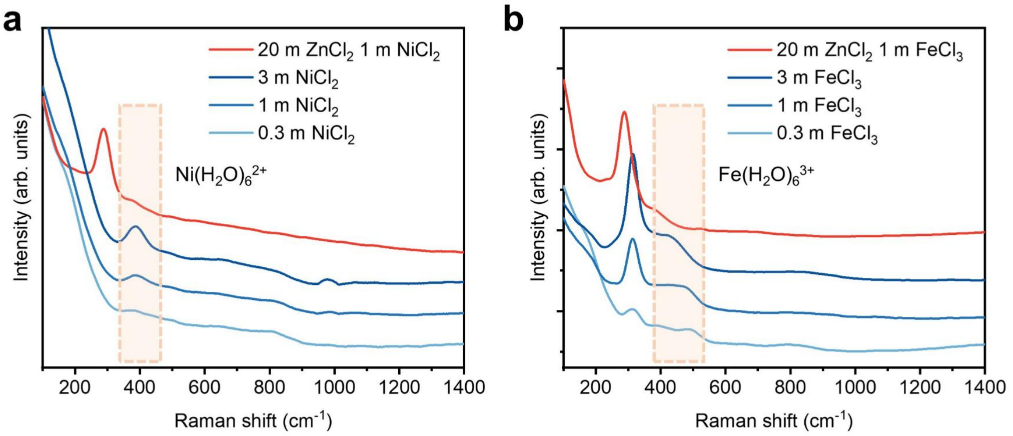

Supplementary Fig. 19 The Raman spectra of **(a)** bare $NiCl_2$ aqueous and $NiCl_2$ in 20 m $ZnCl_2$ aqueous solution, **(b)** bare $FeCl_3$ aqueous and $FeCl_3$ in 20 m $ZnCl_2$ aqueous solution.

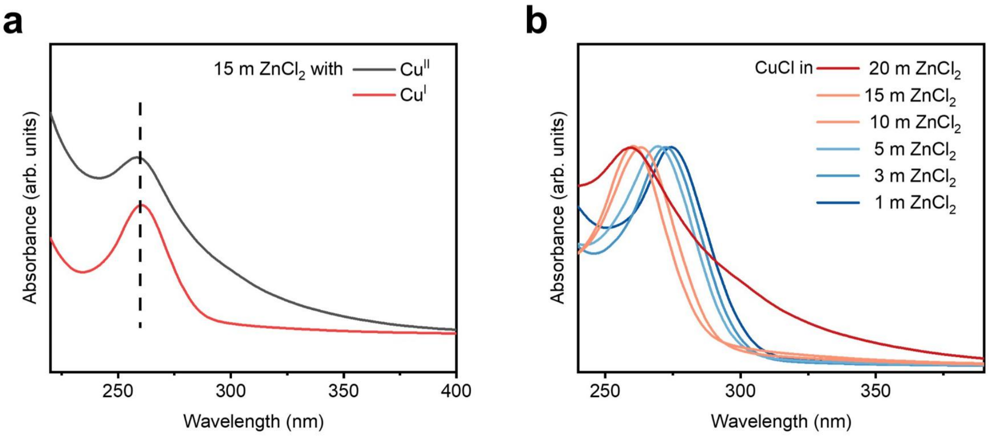

Supplementary Fig. 20 The UV-vis absorbance of **(a)** cupric and cuprous ion in 15 m $ZnCl_2$, **(b)** cuprous ion in various of $ZnCl_2$ solutions.

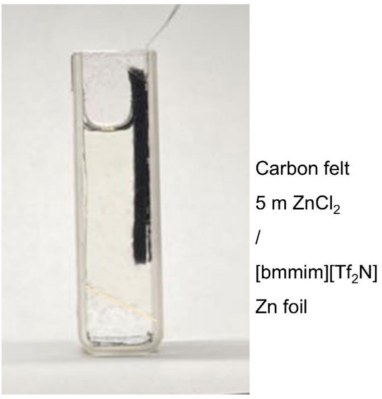

Supplementary Fig. 21 The optical photograph of the homemade quartz electrochemical cell.

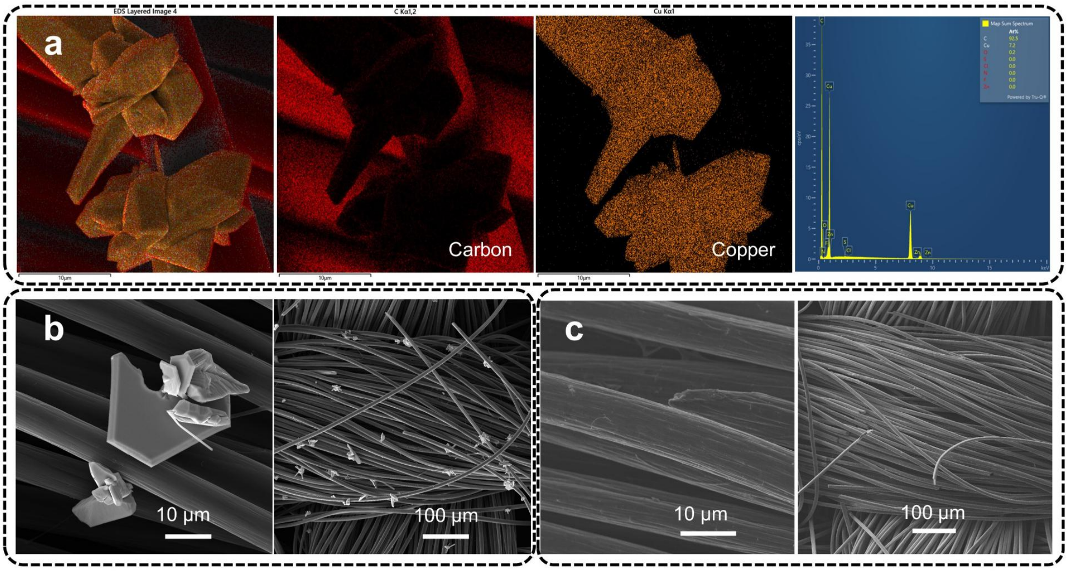

Supplementary Fig. 22 (a) The EDS mapping of Cu deposits on carbon cloth. The SEM images of carbon cloth **(b)** discharged to 0.4 V and **(c)** charged to 1 V.

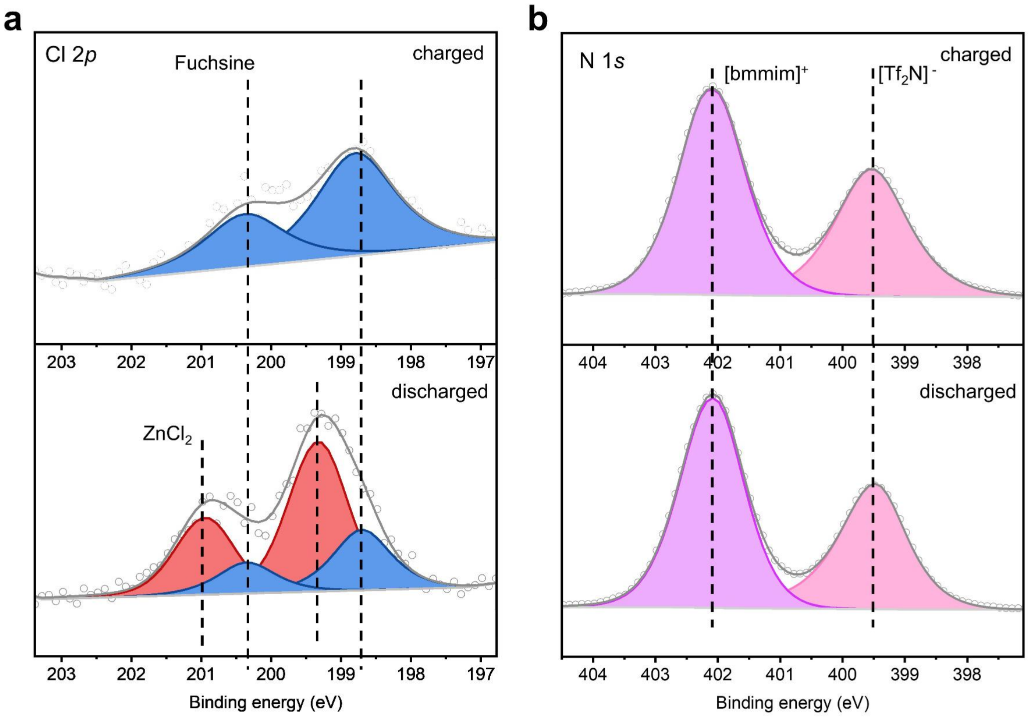

Supplementary Fig. 23 High-resolution XPS analysis of the zinc anodes at charged and discharged states, (a) Cl 2*p* and (b) N 1*s*.

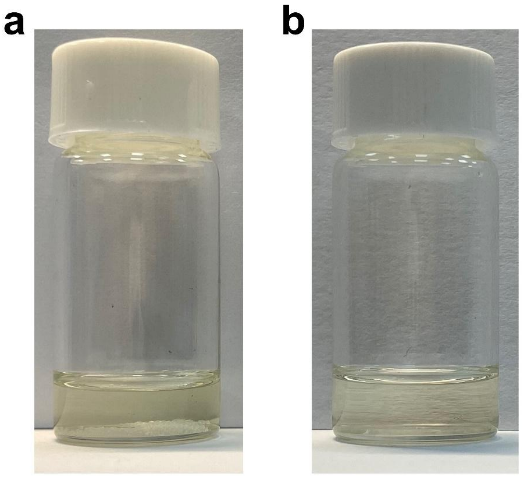

Supplementary Fig. 24 (a) The optical photographs of 0.125 mmol $ZnCl_2$ and **(b)** 0.125 mmol $Zn(Tf_2N)_2$ in the [bmmim][Tf_2N] ionic liquid (1 mL) to show their solubility.

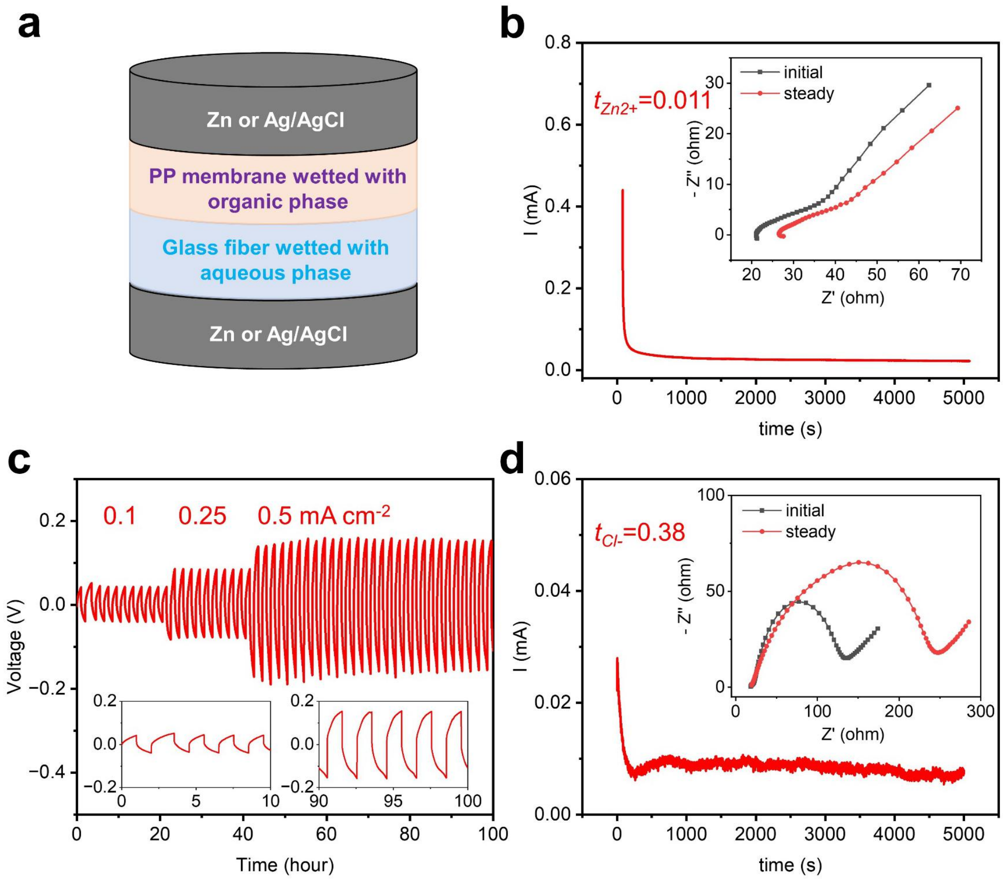

Supplementary Fig. 25 (a) Schematic diagram of the cells used for the chronoamperometry test. The biphasic electrolyte was consisted of 30 μL aqueous phase and 10 μL organic phase. **(b)** CA curves of Zn//Zn cell based on the bi-phasic electrolyte with a perturbation potential of 20 mV, and the insets are the electrochemical impedance spectra at initial and steady states, respectively. **(c)** The galvanostatic Cl^- absorption/extraction reactions in Ag/AgCl//Ag/AgCl cell based on the bi-phasic electrolyte at 0.1 mAh cm^{-2}, 0.25 mAh cm^{-2}, and 0.5 mAh cm^{-2}. Ag/AgCl electrode was prepared by mixing 20 wt% Super P carbon, 70 wt% AgCl, 10 wt% PVDF, with NMP as the solvent. The slurry was cast onto the Ag foam (mg cm^{-2}) and was vacuum dried at 60 °C for 12h. The areal loading of AgCl is about mg·cm^{-2} for each electrode. **(d)** The CA curves of Ag/AgCl//Ag/AgCl cell under the same condition as that of Zn//Zn cell.

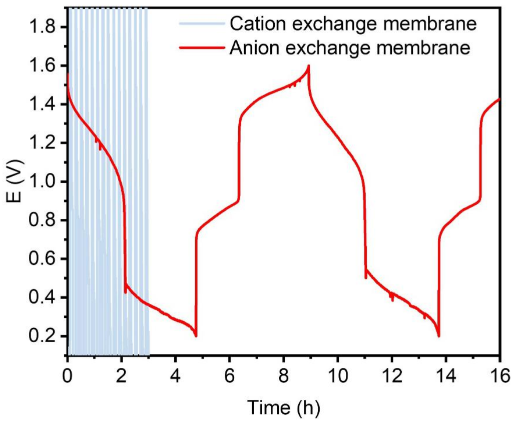

Supplementary Fig. 26 Voltage profiles of the Zn-Cu battery based on the bi-phasic electrolyte with an anion or cation exchange membranes at 80 mAh g^{-1}.

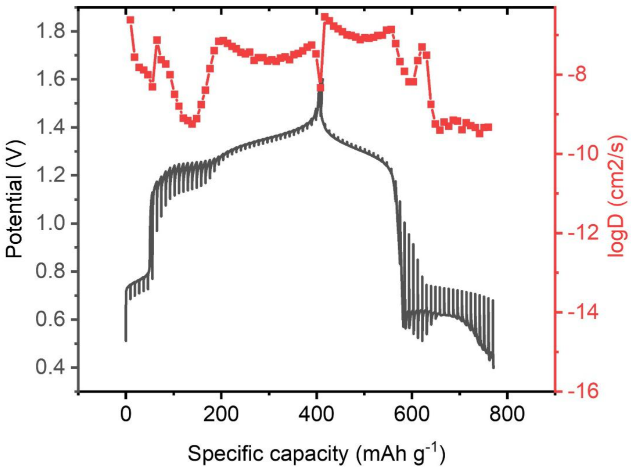

Supplementary Fig. 27 The diffusion coefficient and the voltage profiles of Zn-Cu battery during GITT measurement. The diffusion coefficient D in the active material can be estimated according to the following equation:

$$D = \frac{4L^2}{\pi\tau} \times (\frac{\Delta Es}{\Delta Et})^2 \qquad (1)$$

Where τ is the constant current pulse duration; L is the ion diffusion distance (cm), and the cathode thickness is used to express its value; ΔE_s and ΔE_t are the change in the steady state voltage and overall cell voltage after the application of a current pulse in a single step GITT experiment, respectively.

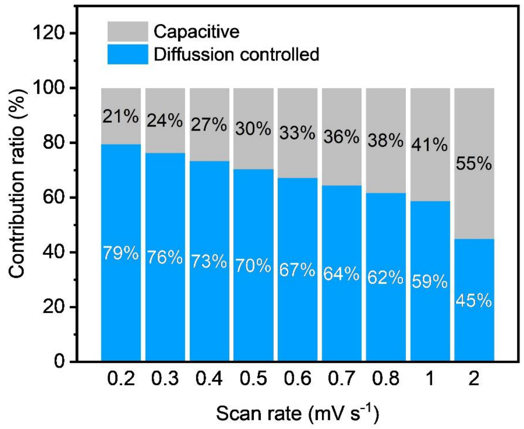

Supplementary Fig. 28 The capacitive contribution to total capacity of the Zn-Cu battery at different scan rate. The percentage of capacitive contribution is quantitatively analyzed by the following equation:

$i(V) = k_1\nu + k_2\nu^{1/2}$ (2)

Where k_1 and k_2 are defined constants. $k_1\nu$ represents the capacitive-controlled contribution and $k_2\nu^{1/2}$ refers to the diffusion-controlled contribution.

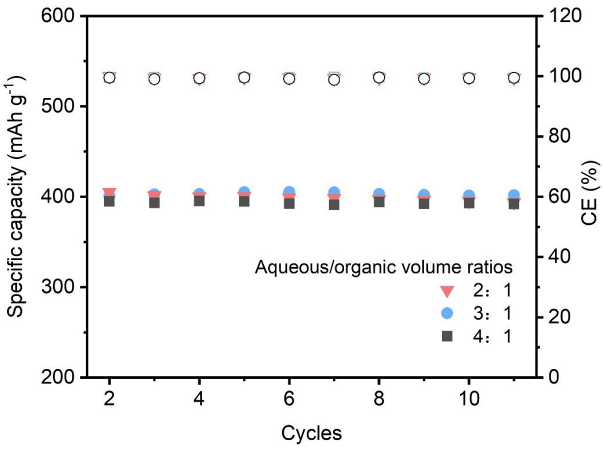

Supplementary Fig. 29 Cycling performance of the Zn-Cu batteries in biphasic electrolytes with different volume ratios between aqueous phase and organic phase. The volume of the organic phase was kept at 10 μL.

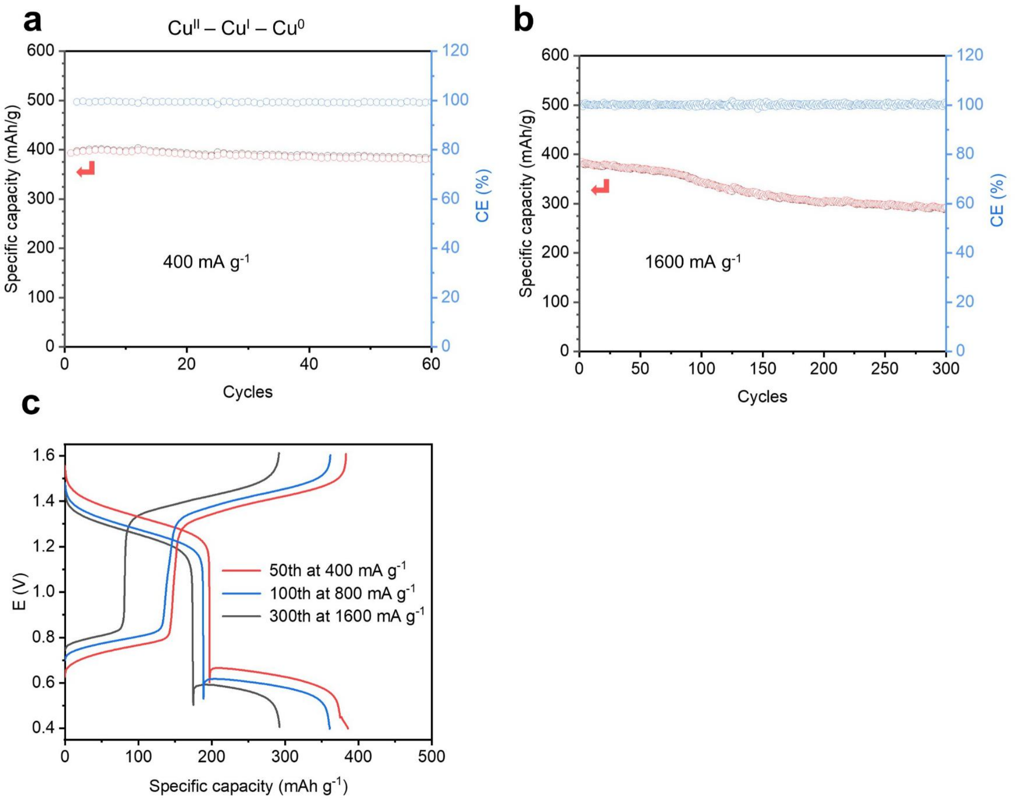

Supplementary Fig. 30 Cycling performance of the Zn-Cu cell between 1.6 – 0.4 V at **(a)** 400 and **(b)** 1600 mA g^{-1}**.** **(c)** The voltage profiles of the Zn-Cu cells.

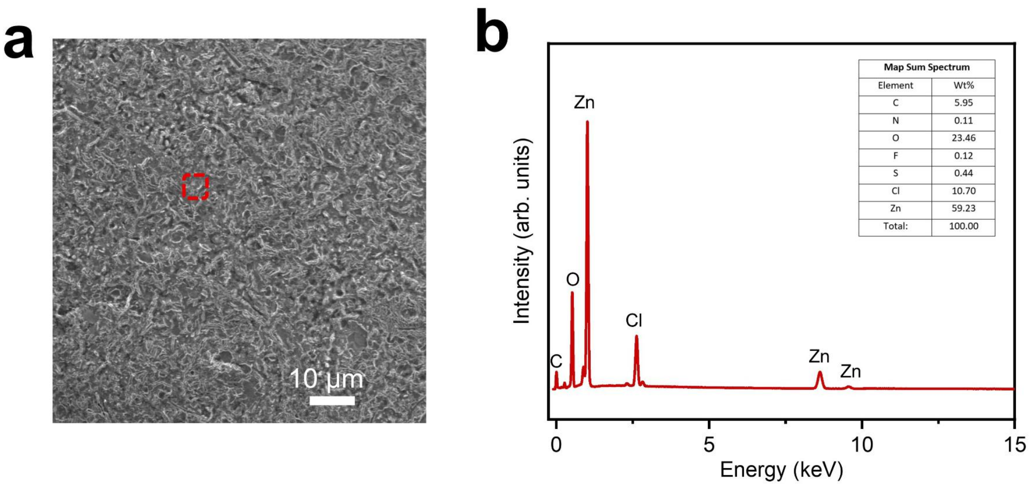

Supplementary Fig. 31 (a) The SEM images of Zn anode after 100 cycles in 15 m $ZnCl_2$ biphasic electrolyte, at 800 mAh g^{-1} between 0.4 – 1.6 V, and **(b)** EDS elemental analysis for the selected area in (a).

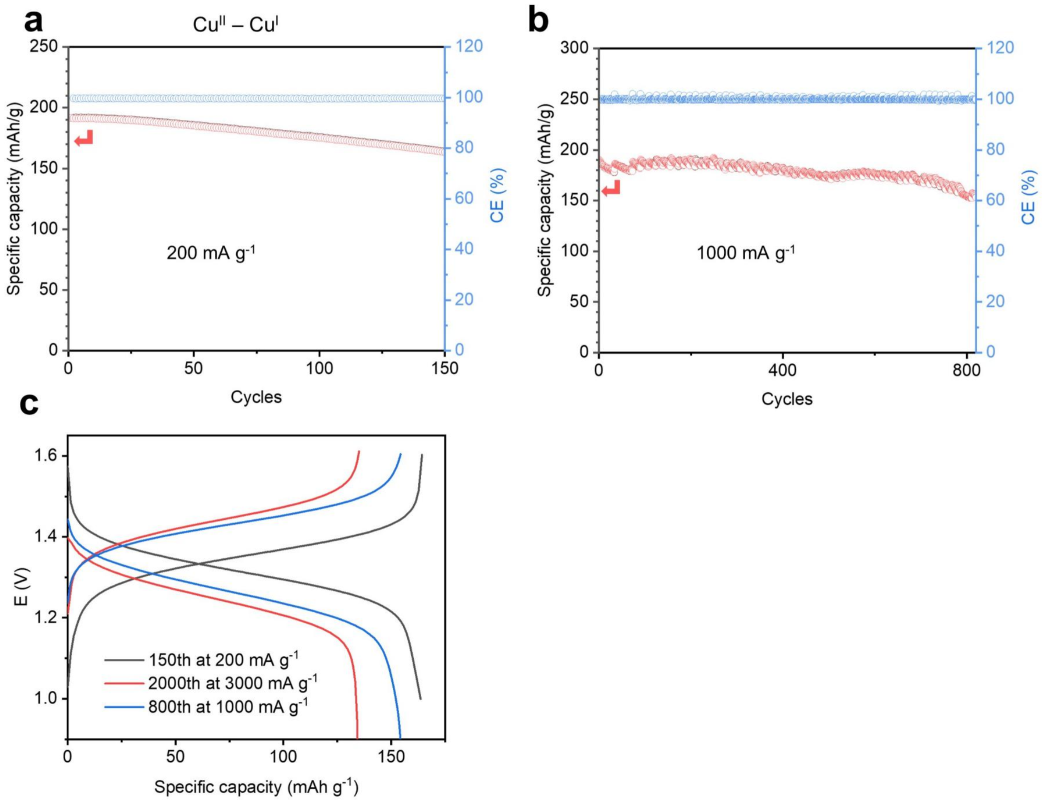

Supplementary Fig. 32 Cycling performance of the Zn-Cu cell between **(a)** 1.6 – 1.0 V at 200 mA g^{-1} and **(b)** 1.6-0.9 V at 1000 mA g^{-1}**. (c)** The corresponding voltage profiles.

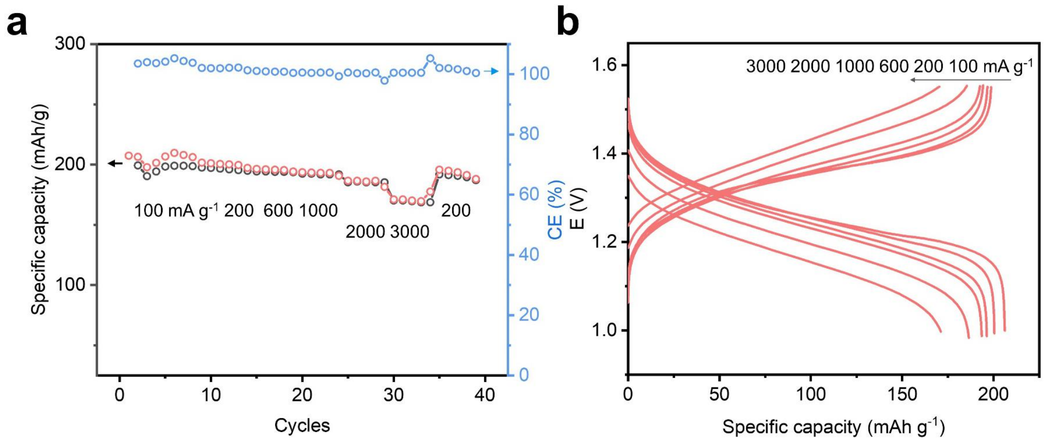

Supplementary Fig. 33 Rate capability of the Zn-Cu battery at current densities ranging from 100 to 3000 mA g^{-1}.

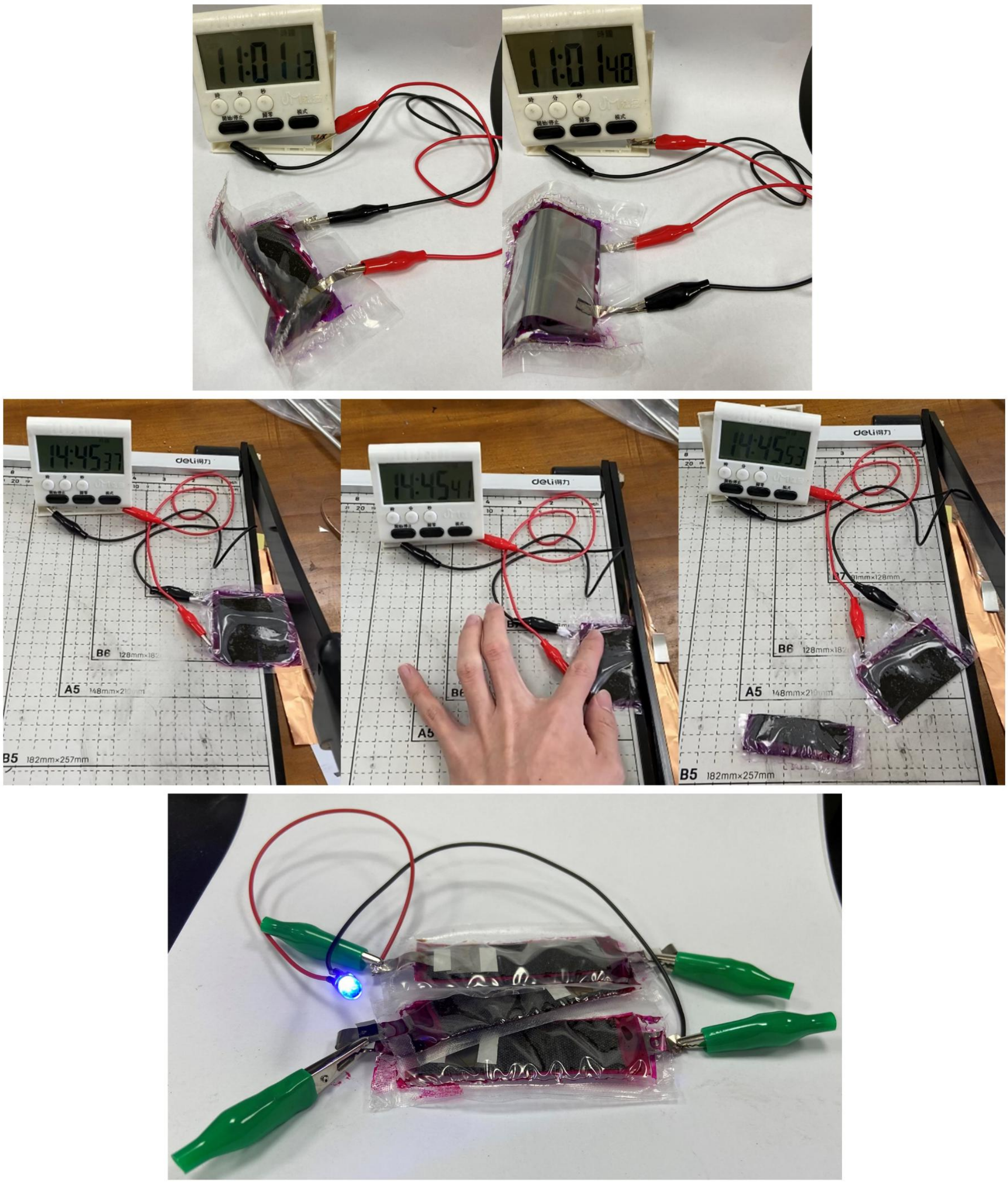

Supplementary Fig. 34 The digital images of the bending and cutting experiment of the flexible Zn-Cu battery. The pouch cell was cut into three pieces, and was connected in series to light up a 3V blue LED lamp.

Supplementary Note 1

The pictures of the electrodes and separators are shown in **Supplementary Fig. 35**. Since the biphasic electrolyte is fluidity for both phases, it is more facile to use the biphasic electrolyte by employing absorbents in the battery to prevent any distribution of the separation. The hydrophobic PP membrane was used as the absorbent for the organic phase (**Supplementary Fig. 36a**), while the hydrophilic glass fiber was used for the aqueous phase (**Supplementary Fig. 36b**). It also shows that the PP membrane could not be wetted by the aqueous phase (**Supplementary Fig. 36c**), whereas both the glass fiber and carbon cloth could be wetted (**Supplementary Fig. 36d, e**). The contact of these two absorbent layers in the battery could form the biphasic interface automatically. The battery was assembled by simply stacking the electrodes and the wetted separators layer by layer (**Supplementary Fig. 37a, b**). The complete cell configurations for the Swagelok cell and pouch cell before sealing are shown in **Supplementary Fig. 37c, d, e**. The cells were vertically placed with the anode side (organic phase) on the top during the electrochemical test.

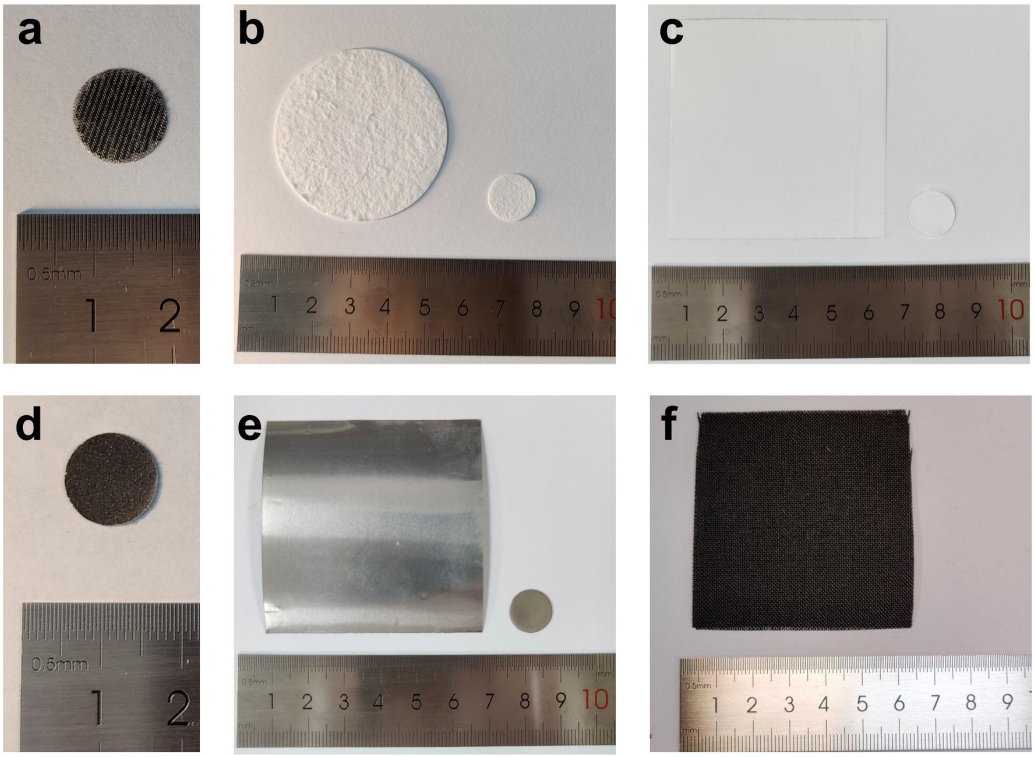

Supplementary Fig. 35 Pictures of the **(a)** Ti mesh supported $CuCl_2$ cathode, **(b)** glass fiber separator, **(c)** PP separator, **(d)** Ag/AgCl electrode, **(e)** zinc anode, and **(f)** carbon cloth.

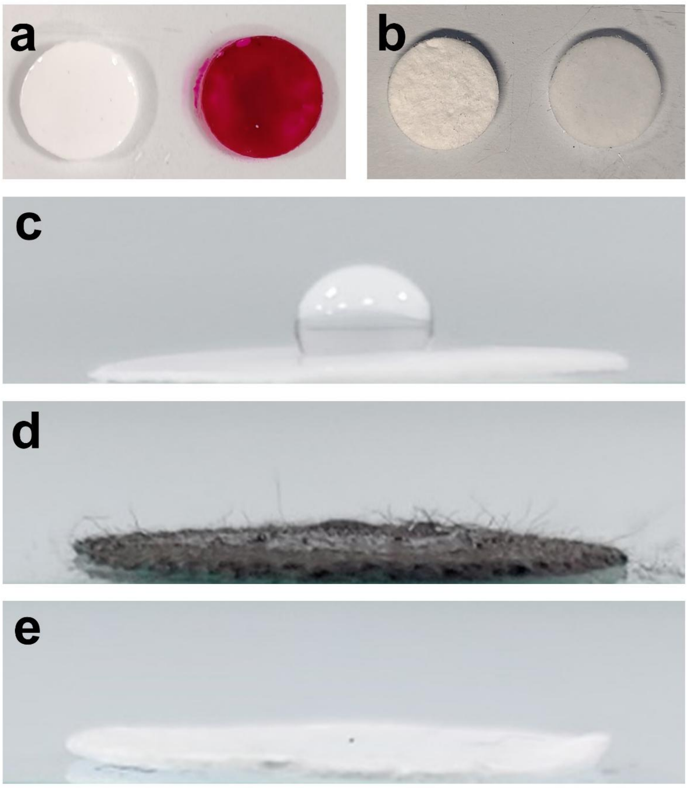

Supplementary Fig. 36 Pictures of the **(a)** PP separator, PP separator absorbed with the organic phase, **(b)** glass fiber, and glass fiber wetted by the aqueous phase. Note that the organic phase turned red color after 0.25 m fuchsine was added. Pictures of the **(c)** PP separators, **(d)** carbon cloth, and **(e)** glass fiber with the contact of 10 μL aqueous electrolyte.

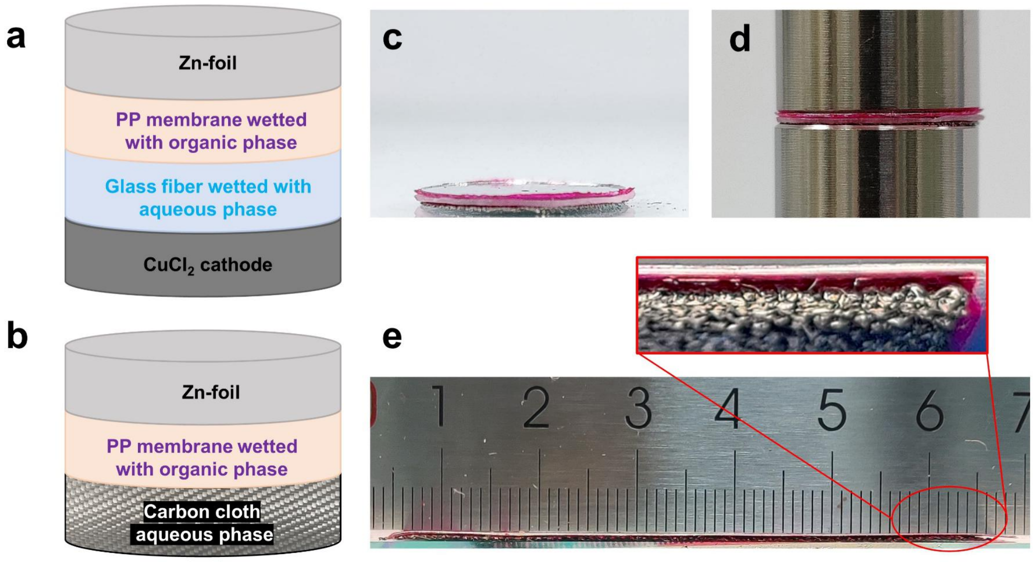

Supplementary Fig. 37 **(a)** and **(b)** Schematic diagrams for the proposed Zn-Cu battery and flexible pouch cell, respectively. **(c)**,**(d)** a complete cell before sealing in Swagelok cell case. **(e)** the flexible pouch cell before sealing.

Table S1 Comparison of various batteries with Cu chemistry. Ion-exchange membrane is denoted as IEM. The areal capacities are given in square brackets [].

Cathode reaction and output voltage	Capacity ($mAh\ g^{-1}$) based on m_{Cu}	Active materials utilization (current density based on m_{Cu})	Cycle life	Capacity retention	Overpotential (V)	Ref
$2Cu^{2+} + 2e^- \rightarrow Cu^0$; 0.9 V;	165 [7.25 $mAh\ cm^{-2}$]	19% at 2 $mA\ cm^{-2}$ (0.05C)	Primary battery	/	/	1
$Cu^{2+} + 2e^- \rightarrow Cu^0$; 0.96 V; IEM supported	330 [/]	39% at 1 $mA\ cm^{-2}$	/	/	0.3	2
$Cu^{2+} + 2e^- \rightarrow Cu^0$; 0.8 V; IEM supported	763 [0.5 $mAh\ cm^{-2}$]	90% at 0.5 $mA\ cm^{-2}$ (0.5C)	100	95%	0.35	3
$Cu(OH)_2/CuO + 2e^- \rightarrow Cu^0$; 0.76 V	718 [1.5 $mAh\ cm^{-2}$]	85% at 0.12C	200	55.8%	0.45	4
$CuO + 2e^- \rightarrow Cu^0$; 0.75 V	843 (674 based on m_{CuO}) [81 $mAh\ cm^{-2}$]	>99% at 0.2C	150	56.0%	0.3	5
$CuO + 2e^- \rightarrow Cu^0$; 0.8 V	550 [/]	66% at 0.2C	200	54.5%	0.3	6
$Cu_2CO_3(OH)_2 + 4e^- \rightarrow Cu^0$; 0.4 V	665 [13.3 $mAh\ cm^{-2}$]	81% at 0.6C	50	48.9%	0.3	7
$Cu^{II} + e^- \rightarrow Cu^I$; 1.3 V<br>$Cu^I + e^- \rightarrow Cu^0$; 0.7 V	814 (385 based on m_{CuCl2}) [2 $mAh\ cm^{-2}$]	96% at 2C	100	93.5%	0.15	This work
	791 (374 based on m_{CuCl2}) [1.9 $mAh\ cm^{-2}$]	94% at 4C	300	76.0%	0.2	
$Cu^{II} + e^- \rightarrow Cu^I$; 1.3 V	397 (188 based on m_{CuCl2}) [1 $mAh\ cm^{-2}$]	94% at 2.5C	800	80.9%	0.15	This work
	370 (175 based on m_{CuCl2}) [0.9 $mAh\ cm^{-2}$]	88% at 7.5C	2000	80.2%	0.2	

References

1. Duan, J. *et al.* Tough hydrogel diodes with tunable interfacial adhesion for safe and durable wearable batteries. *Nano Energy* **48**, 569–574 (2018).

2. Zhang, H. *et al.* Using Li^+ as the electrochemical messenger to fabricate an aqueous rechargeable Zn–Cu battery. *Chem. Commun.* **51**, 7294–7297 (2015).

3. Jameson, A., Khazaeli, A. & Barz, D. P. J. A rechargeable zinc copper battery using a selective cation exchange membrane. *J. Power Sources* **453**, 227873 (2020).

4. Zhu, Q. *et al.* Realizing a rechargeable high-performance Cu-Zn battery by adjusting the solubility of Cu^{2+}. *Adv. Funct. Mater.* **29**, 1–8 (2019).

5. Schorr, N. B. *et al.* Rechargeable alkaline zinc/copper oxide batteries. *ACS Appl. Energy Mater.* **4**, 7073–7082 (2021).

6. Arnot, D. J., Schorr, N. B., Kolesnichenko, I. V. & Lambert, T. N. Rechargeable alkaline Zn–Cu batteries enabled by carbon coated Cu/Bi particles. *J. Power Sources* 529, 231168 (2022).

7. Gallagher, T. C. et al. From Copper to Basic Copper Carbonate: A Reversible Conversion Cathode in Aqueous Anion Batteries. *Angew. Chemie Int. Ed.* (2022) doi:10.1002/anie.202203837.
